# Acute Mesenteric Vein Thrombosis in a Pregnant Patient at 10 Weeks Gestation: A Case Report

**DOI:** 10.3390/diagnostics11081348

**Published:** 2021-07-27

**Authors:** Ying-Ying Chen, Sheng-Mao Wu, Russell Oliver Kosik, Yi-Chien Hsieh, Tzu-I Wu, Wing P. Chan

**Affiliations:** 1 Department of Radiology, Wan Fang Hospital, Taipei Medical University, Taipei 11031, Taiwan; cii411@gmail.com (Y.-Y.C.); wingchan@tmu.edu.tw (W.P.C.); 2 Department of Radiology, School of Medicine, College of Medicine, Taipei Medical University, Taipei 11031, Taiwan; russkosik1@hotmail.com; 3 Division of Trauma and Emergency Surgery, Department of Surgery, Wan Fang Hospital, Taipei Medical University, Taipei 11031, Taiwan; mitralwu@hotmail.com; 4 Department of Obstetrics and Gynecology, Wan Fang Hospital, Taipei Medical University, Taipei 11031, Taiwan; sharah4910@tmu.edu.tw; 5 Department of Obstetrics and Gynecology, School of Medicine, College of Medicine, Taipei Medical University, Taipei 11031, Taiwan

**Keywords:** acute abdomen, computed tomography (CT), mesenteric vein thrombosis, magnetic resonance imaging (MRI), ischemic bowel disease, pregnancy

## Abstract

Acute abdominal pain during pregnancy is challenging, both from a diagnostic and management perspective. A non-localized, persistent pain out of proportion to physical examination is a sign that advanced imaging may be necessary. Mesenteric venous thrombosis in a pregnant patient is extremely rare, but if diagnosis is delayed, can be potentially fatal to both the mother and the fetus. We present here a pregnant patient in the tenth week of gestation with classic clinical manifestations of mesenteric vein thrombosis and the corresponding findings on magnetic resonance imaging (MRI) and computed tomography (CT).

## 1. Introduction

Mild abdominal pain during pregnancy is common, but intense pain can be a sign of a serious problem [1]. Some etiologies require immediate treatment, as they can be life-threatening to both the mother and the fetus. Some of these conditions include acute appendicitis, cholecystitis, ectopic pregnancy [2,3], ovarian torsion [4], and deep vein thrombosis [5]. Evaluation of abdominal pain can prove a diagnostic challenge during pregnancy. There are roles for ultrasound, MRI, and even CT, according to the ACR Appropriateness Criteria. 

## 2. Case Presentation 

A 33-year-old gravida 2, para 1, female presented to our emergency department in her tenth week of gestation complaining of epigastric pain for 2 days that did not improve, despite medical treatment at an outside hospital. She denied significant medical history. She also reported nausea, vomiting, poor appetite, and abdominal distension. Upon arrival, she was tachycardic (105 bpm), but she had no fever, hypotension, or tachypnea. Physical examination revealed epigastric and suprapubic tenderness though no rebound pain. Pertinent laboratory examinations included leukocytosis (28 × 100/uL with left shift (neutrophils, 95%), elevation of C-reactive protein (8.7 m/dL), and elevation of Glutamic-Pyruvate Transaminase (138 U/I).

**Figure 1:**
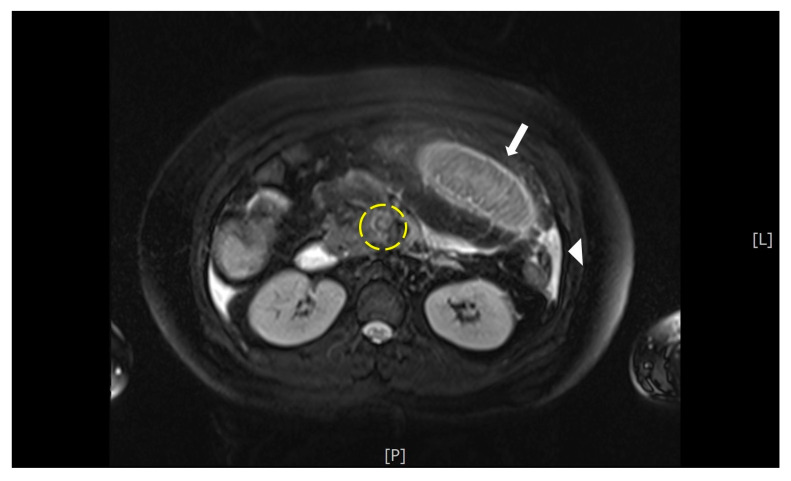
MRI This T2- fat saturation image demonstrates a hyperintense filling defect within the superior mesenteric vein (yellow circle). There is also thickening of the wall of a small bowel loop (arrow), and a small amount of ascites (arrow head).

**Figure 2:**
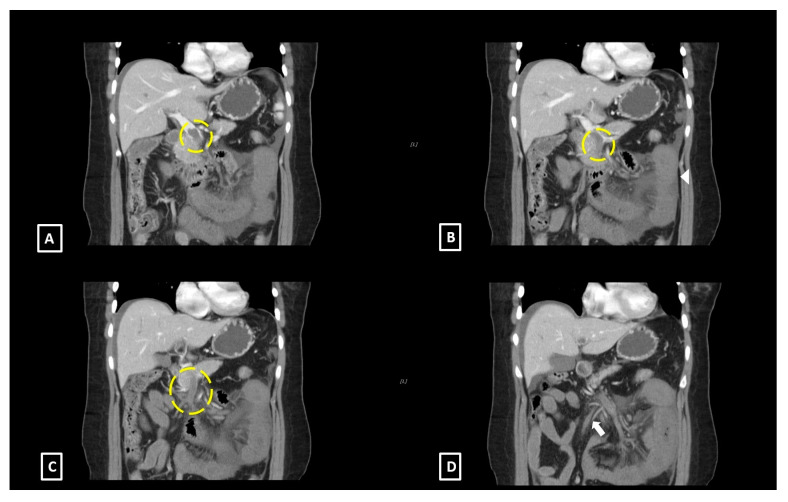
Coronal computed tomography images of the abdomen with intravenous contrast demonstrate: (**A**) A large filling defect within the main portal vein (yellow circle). (**B**) A large filling defect within the superior mesenteric vein (yellow circle) as well as circumferential thickening and hypoenhancement of the small bowel wall. (**C**) Extension of the thrombus to the superior mesenteric vein inferiorly and its branches (yellow circle). (**D**) Engorged superior mesenteric venous branches inferior to the thrombus (arrow).

Abdominal ultrasonography revealed a living 10-week-old fetus, mild maternal ascites, and no sonographic evidence of acute cholecystitis or appendicitis. Magnetic resonance imaging (MRI) was performed and demonstrated a T2 hyperintense filling defect within the superior mesenteric vein (SMV), small bowel wall thickening, and ascites (Figure 1). With a suspected diagnosis of bowel ischemia secondary to SMV thrombosis, contrast-enhanced abdominal and pelvic CT (BrightSpeed, General Electric Medical System, Milwaukee, WI, USA), with 5 mm slice thickness, following administration of intravenous contrast was subsequently performed. The CT images demonstrated a filling defect within the main portal vein that extended inferiorly into the SMV and its branches as well as venous engorgement of the SMV branches (Figure 2). Additional findings included circumferential small bowel wall thickening and hypoenhancement as well as edematous changes within the adjacent mesenteric fat. 

**Figure 3:**
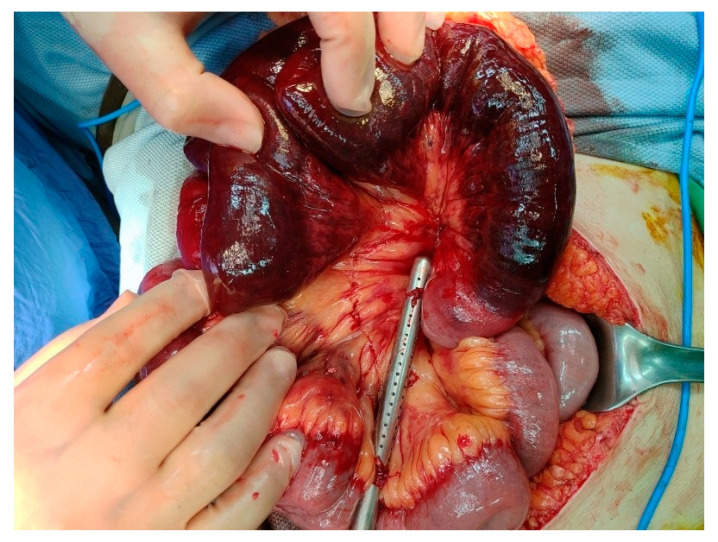
Laparotomy view shows ischemic bowel loop 10 cm distal to the ligament of Treitz.

The patient then underwent emergent diagnostic laparoscopy which, following the observation of ischemic changes to the proximal small bowel and dark red ascites, was converted to laparotomy. Segmental resection of the ischemic bowel with side-to-side anastomosis was performed (Figure 3). The resected small bowel was 70 cm in length, beginning 10 cm distal to the ligament of Treitz. Postoperatively, the patient was transferred to the intensive care unit, where she received total parenteral nutrition, intravenous antibiotics including ceftriaxone and metronidazole, and anticoagulation with enoxaparin. 

Her condition improved, and she was able to resume an oral diet on postoperative day 7. During her admission, a hematologist was consulted to assess for hypercoagulability. The patient’s coagulation profile revealed low levels of antithrombin III (64%) and free protein S antigen (49%). Follow up abdominal CT on postoperative day 8 showed that the thrombus had decreased in size. After discussion with the patient, the decision was made to terminate the pregnancy, and she underwent dilation and curettage on postoperative day 10. She was discharged uneventfully on postoperative day 13, continuing anticoagulation therapy with daily rivaroxaban (15 mg). The patient regularly followed up at both our obstetrics and cardiovascular clinics, with complete resolution of the thrombus noted on follow-up abdominal CT one year later. 

## 3. Discussion

Our patient presented with intractable epigastric pain for 2 days, nausea, and vomiting. Physical examination revealed tachycardia and tenderness over the upper abdomen though without rebound pain. This pain, out of proportion to physical examination, is a warning sign of potentially serious pathology, and an indication for the use of advanced imaging. MRI is therefore an appropriate subsequent imaging tool in pregnant patients who present in this manner. In this case, the definitive diagnosis of mesenteric venous thrombosis was ultimately obtained on CT. 

Mesenteric venous thrombosis in pregnant patients is extremely rare. To the best of our knowledge, less than 20 cases have been reported in the literature [6]. While it is known that pregnancy, due to a combination of physiologic changes that affect coagulation factors and blood flow stasis secondary to inferior vena cava compression by the enlarged uterus, puts patients in a hypercoagulable state [7], because of the paucity of cases, the precise pathogenesis behind the formation of mesenteric venous thromboses during pregnancy is largely unknown. However, the effects are potentially devastating to both the mother and the fetus. Among 15 cases reported by Guan et al, 40% (6/15) ultimately resulted in fetal death or abortion, and one in maternal death [6]. With acute occlusion of mesenteric veins, marked elevations in capillary pressures occur, which lead to bowel wall edema, submucosal hemorrhage, and transmural bowel infarction [8]. Without timely diagnosis and treatment, this eventually results in septic shock and death.

Clinical signs and symptoms of mesenteric venous thrombosis include abdominal pain, abdominal distention, nausea, and vomiting, which are notoriously nonspecific [9,10,11]. Laboratory findings, such as leukocytosis and elevated C-reactive protein, are also fairly nonspecific in working up abdominal pain [9,11]. Complicating matters further is the fact that abdominal pain is a frequent antepartum complaint and is in itself a diagnostic challenge [1]. Factors that contribute to the difficulty of evaluating antepartum abdominal pain include the displacement of abdominal and pelvic structures from their normal locations by the gravid uterus, which makes abdominal examination relatively unreliable, and the physiological leukocytosis that occurs during pregnancy, which can mask the inflammatory response of the underlying pathology [1]. More importantly, acute abdominal pain in pregnant women tends to be non-localized and disproportionate to the physical findings, often persisting beyond two to three hours [12]. Therefore, imaging is essential to ensuring timely and accurate diagnosis. 

Imaging can offer definitive diagnosis of mesenteric venous thrombosis [13]. In non-pregnant patients, CTA offers high accuracy for the diagnosis of acute mesenteric ischemia, with reported sensitivities and specificities as high as 93% to 100% and the potential ability to improve patient survival [14]. However, the high accuracy of CTA is likely because the majority of mesenteric ischemia is due to arterial occlusion rather than venous occlusion. Venous phase abdominal CT for the diagnosis of mesenteric ischemia has been less well studied. Kirkpatrick et al. reported that the addition of arterial phase imaging influenced care in 19% of patients, compared to portal venous phase imaging alone [15]. The presence of mesenteric venous filling defects, which appear as central areas of low attenuation within an enhanced vessel, are the cardinal findings of mesenteric venous thrombosis [10]. Other suggestive though less specific findings include engorgement of mesenteric veins, mesenteric edema, and the presence of collateral vessels [13]. In addition, circumferential bowel wall thickening and abnormal bowel wall enhancement are the most common signs of bowel ischemia [13]. Further, the presence of intraperitoneal fluid carries a poor prognosis, as it may indicate progression to bowel infarction [13]. However, despite the advantages of abdominal CT, the radiation dose imposed upon the fetus is a concern. Therefore, the benefits and risks associated with CT should be discussed with the patient prior to obtaining imaging.

Due to its lack of ionizing radiation, MRI has always been considered one of the most important diagnostic tools for abdominal pain in the pregnant patient. Previous reports have shown that MRI is accurate for the diagnosis of mesenteric thrombosis, with some studies reporting nearly 100% sensitivity and specificity for MR angiography (MRA) [16]. One of the advantages of MRI is its ability to determine the age of the thrombus [13]. Acute to subacute thrombi, less than 5 weeks old, are likely to have higher signal intensity compared to the liver on T1 and T2-weighted sequences, whereas chronic thrombi tend to show a significant decrease in signal intensity on T1-weighted sequences [13]. MRI may also offer evidence of the potential cause of the thrombus formation, such as acute pancreatitis, cirrhosis, or hepatocellular carcinoma, and can also rule in or out other causes of antepartum abdominal pain [13,16]. MRA, especially with gadolinium, with its capacity to better depict the vascular structures in the abdomen, provides even higher diagnostic accuracy compared to conventional MRI [16]. However, the use of gadolinium in pregnant patients has remained controversial for decades and is to be generally avoided if clinically feasible in Taiwan. Our case is a wonderful example of the merits of non-contrast MRI in the diagnosis of mesenteric venous thrombosis and a reminder that the diagnosis can often be made or at least inferred without the administration of gadolinium.

Alternatively, the application of Doppler ultrasound can be a decisive examination in diagnosing SMV thrombosis. Diagnosis can be confidently established if the obstruction of the portal or mesenteric vein by an intraluminal echogenic lesion shown on ultrasound. Color flow Doppler ultrasound has an excellent sensitivity (100%) and specificity (93%) in detecting portal and mesenteric vein thrombosis [13]. Other accompanied sonographic findings may include ascites, splenomegaly, intestinal congestions, and extensive venous collaterals [17]. However, the role of ultrasound imaging in diagnosing mesenteric venous thrombosis in a pregnant patient was unclear.

Treatment of mesenteric venous thrombosis depends on the severity of the patient’s condition. If there are no signs of bowel infarction, the patient can be managed conservatively with fluid resuscitation, broad-spectrum antibiotics, bowel rest and, most importantly, systemic anticoagulation [9]. However, surgery should not be delayed if bowel necrosis or perforation are suspected, and in such cases immediate exploration with either an open or laparoscopic approach should be performed. The infarcted segment of bowel should be adequately resected and the adjacent segments can be directly anastomosed. Some advocate catheter-based thrombolysis or mechanical thrombectomy as an adjuvant to anticoagulation, though the current evidence is not yet adequate to definitively support its use [9]. The cause of the hypercoagulability should be identified following emergent management so as to determine the future use of anticoagulation [9]. Special consideration in pregnant patients is warranted when certain diagnostic or treatment techniques may affect the gestation. Previous reports have shown that vaginal delivery of a normal baby is possible even when mesenteric venous thrombosis occurs at an early gestational age [6], though careful evaluation is necessary, and as always, continued communication with the patient is vital.

In summary, we present the rare case of a pregnant 33-year-old female who experienced mesenteric vein thrombosis and ischemic bowel in her tenth week of gestation. Timely and accurate diagnosis in such a scenario requires a high-index of clinical suspicion, and through this case we highlight the importance of early imaging, especially conventional MRI.

## Data Availability

All data are available within the article.
